# Infiltrated plaques resulting from an injury caused by the common octopus (*Octopus vulgaris*): a case report

**DOI:** 10.1186/1678-9199-20-47

**Published:** 2014-10-24

**Authors:** Vidal Haddad Jr, Claudia Alves de Magalhães

**Affiliations:** Department of Dermatology, Botucatu Medical School, São Paulo State University (UNESP – Univ Estadual Paulista), Caixa Postal 557, Botucatu, 18.618-000 São Paulo State Brasil; Coordination for Ocean Affairs and Antarctica, Brazilian Ministry of Science, Technology and Innovation, Brasília, DF Brasil

**Keywords:** Octopuses, Venomous animals, Aquatic animals, Venoms, Poisoning, Digestive enzymes

## Abstract

Several species of octopus are considered venomous due to toxins present in the glands connected to their “beak”, which may be associated with hunt and kill of prey. Herein, we report an accident involving a common octopus (*Octopus vulgaris*) that injured an instructor during a practical biology lesson and provoked an inflamed infiltrated plaque on the hand of the victim. The lesion was present for about three weeks and was treated with cold compresses and anti-inflammatory drugs. It was healed ten days after leaving a hyperchromic macule at the bite site. The probable cause of the severe inflammation was the digestive enzymes of the glands and not the neurotoxins of the venom.

## Background

Cephalopod mollusks are marine animals that include squids, octopuses, cuttlefish, and nautiluses. Octopuses have eight feet and a horny beak that is used to capture prey and for defense strategies including jets of water that propel their bodies quickly in the opposite direction of perceived threats and ejection of clouds of dark ink to confuse predators [1]. The suckers on their arms are capable of provoking purpuric lesions by suction, whereas their beaks can inflict lacerations to victims (especially fishermen and divers), where the venom contained in their salivary glands penetrates the body [1–3]. The venom contains digestive enzymes and proteinaceous neurotoxins that immobilize prey. *Octopus vulgaris* is the most common octopus found off the Brazilian and South American coast and recent reports have indicated the presence of cephalotoxin, a glycoprotein, in the saliva of this species [4]. The consumption of raw octopus may provoke neuromuscular toxicity [4]. Herein, we report an accident involving a common octopus (*Octopus vulgaris*), which injured an instructor in a practical biology lesson, causing an inflamed infiltrated plaque on the hand of the victim. Although there was no initial manifestation besides local pain, the wound had a chronic evolution.

## Case presentation

**Figure 1:**
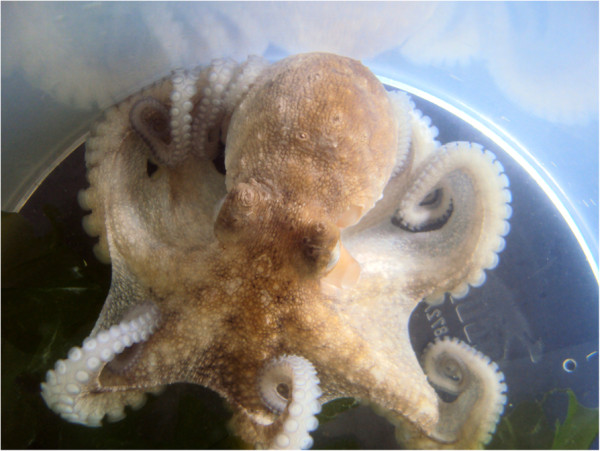
***Octopus vulgaris*** **, this animal caused the injury described in the text.**

**Figure 2:**
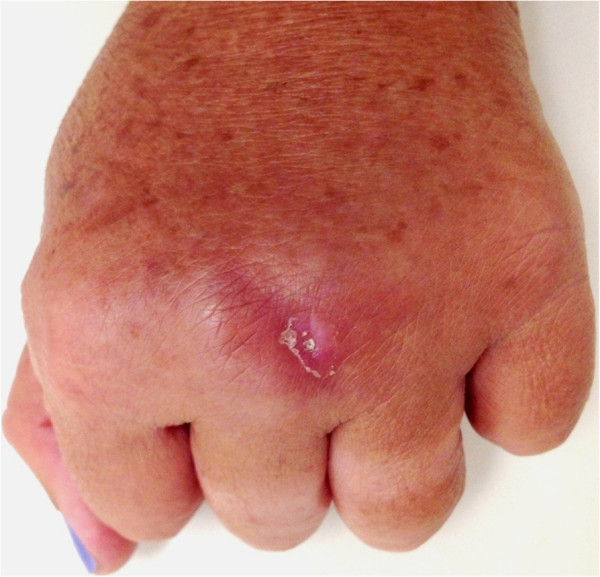
**Chronic infiltrated plaque on the dorsum of the left hand of the patient.** The image shows the central ulceration caused by the beak of the octopus.

The patient, a 53-year old female marine biologist, was “bitten” on the dorsum of the left hand by a small octopus three weeks before seeking medical attention. The injury occurred during a practical biology lesson when the patient was handling the live octopus (Figure 1). An erythematous edematous plaque, about 5.0 cm in diameter, appeared surrounding a small ulcer at the bite site. The wound was initially painful, but the reason for seeking medical help was due to difficulty in healing and the development of infiltration, hard on palpation, giving the site a hardened aspect (Figure 2). The injury was treated with cold compresses and a non-steroidal anti-inflammatory drugs for about ten days before resolution, leaving a local hyperchromic macule. No histopathological exam was performed due to the regression of the lesion when the patient returned after treatment.

Common octopuses (*Octopus vulgaris*) represent the majority of octopuses captured in Brazil [5]. Similarly to Indo-Pacific genera (including *Hapalochlaena*, the blue-ringed octopus), they possess digestive enzymes and neurotoxins in glands connected to their horny beak and may use this venom for defense or to subdue prey. Blue-ringed octopus venom contains mainly tetrodotoxin (like puffer fish venom), while common octopus venom is composed of cephalotoxin, a toxin less powerful than tetrodotoxin, but also capable of causing paralysis and other manifestations in humans [1–4]. The exact effects of the digestive enzymes are not known, but they can clearly provoke inflammatory reactions in victims’ tissue.

## Conclusions

Our patient possibly manifested the effects of the enzymatic action of octopus saliva, and not the neuromuscular signs of toxins from the venom, since tingling in the hand or arm, muscle weakness or systemic manifestations were not reported. Possible complications of envenomations include secondary infections, with fever and purulent secretion. In this case, the response to non-steroidal anti-inflammatory drugs was good and probably abbreviated the evolution of the problem.

## Consent

Consent was obtained from the patient for publication of this case report and accompanying images.
